# Pancreatic Remnant Occlusion after Whipple's Procedure: An Alternative Oncologically Safe Method

**DOI:** 10.1155/2013/960424

**Published:** 2013-08-05

**Authors:** Theodosios Theodosopoulos, Dionysios Dellaportas, Anneza I. Yiallourou, George Gkiokas, George Polymeneas, Alexios Fotopoulos

**Affiliations:** 2nd Department of Surgery, Aretaieion Hospital, University of Athens, 76 Vasilissis Sophias Avenue, 11528 Athens, Greece

## Abstract

*Introduction*. To present our experience regarding the use of pancreatic stump occlusion technique as an alternative management of the pancreatic remnant after pancreatoduodenectomy (PD). *Methods*. Between 2002 and 2009, hospital records of 93 patients who had undergone a Whipple's procedure for either pancreatic-periampullary cancer or chronic pancreatitis were retrospectively studied. In 37 patients the pancreatic duct was occluded by stapling and running suture without anastomosis of the pancreatic remnant, whereas in 56 patients a pancreaticojejunostomy was performed. Operative data, postoperative complications, oncological parameters, and survival rates were recorded. *Results*. 2/37 patients of the occlusion group and 9/56 patients of the anastomosis group were treated for chronic pancreatitis, whereas 35/37 and 47/56 patients for periampullary malignancies. The duration of surgery for the anastomosis group was significantly longer (mean time 220 versus 180 minutes). Mean hospitalization time was 6 days for both groups. The occlusion group had a lower morbidity rate (24% versus 32%). With regard to postoperative complications, a slightly higher incidence of pancreatic fistulas was observed in the anastomosis group. *Conclusions*. Pancreatic remnant occlusion is a safe, technically feasible, and reducing postoperative complications alternative approach of the pancreatic stump during Whipple's procedure.

## 1. Introduction 

Progress in surgical technique and perioperative management has significantly reduced the morbidity and mortality rate of pancreatic resection procedures [1, 2]. The majority of postoperative complications after pancreatoduodenectomy (PD) arise from pancreatic leakage by the pancreatic stump. The pancreatic anastomosis is called by some authors the “Achilles heel” of pancreatic surgery due to its high rate of complications among all abdominal anastomoses [3, 4]. The optimal management of the pancreatic remnant after PD remains a challenge. More than 80 different methods of pancreaticoenteric reconstruction have been described, indicating the absence of a gold standard technique [5]. An interesting alternative option is the pancreatic stump occlusion technique with various methods. Our institution's eight-year experience using this approach in a nonselected group of patients is presented herein. The objective of our trial was to compare the two operative approaches for the management of the pancreatic remnant with regard to mean operative time, postoperative complications, oncological parameters, and one-year survival rates.

## 2. Materials and Methods

A retrospective study was performed in a nonselected series of 93 patients treated between September 2002 and December 2009 with suspected pancreatic and periampullary cancers or chronic pancreatitis. All patients underwent Whipple's procedure in our hospital. There were 65 men (70%) and 28 women (30%), and their mean age was 64 years (range, 41–83 years). The first group included 37 patients, in which the pancreatic duct was occluded by stapling and a running 3–0 polypropylene suture without anastomosis of the pancreatic remnant. The second group composed of 56 patients, in which a traditional end-to-end or end-to-side invagination pancreaticojejunostomy was performed after PD. The pancreatic remnant was invaginated into the jejunum by about 2 cm, and two-layer sutures were performed interruptedly. In all cases one drainage tube was placed posterior to the biliary-jejunal anastomosis, and for the second group the same tube was placed over the pancreatic anastomosis as well. The drainage was removed on the third postoperative day, unless a pancreatic fistula was confirmed. All patients were operated by the same surgical team.

The indications for surgery in the first group were pancreatic cancer (*n* = 26, 70.27%), carcinoma of the Vater's ampulla (*n* = 8, 21.62%), common bile duct carcinoma (*n* = 1, 2.7%), and chronic pancreatitis (*n* = 2, 5.40%) and similarly, in the second group pancreatic cancer (*n* = 34, 60.7%), Vater's ampulla carcinoma (*n* = 12, 21.40%), common bile duct carcinoma (*n* = 6, 10.7%), and chronic pancreatitis (*n* = 4, 7.10%) (Table 1). 

The decision for occlusion of the pancreatic remnant was directed by two criteria:

- pancreatic duct preoperative imaging (either ERCP or MRCP depicting an already occluded duct),
- intraoperative appearance of the duct indicating increased intraductal hydrostatic pressure (spurt of the pancreatic fluid at the time of opening of the pancreatic duct).

## 3. Results

The mean operative time for the occlusion group was 180 minutes (range 160–200 minutes) versus 220 minutes (range 190–240 minutes) in the anastomosis group. Mean hospitalization time was 6 days (range 4–11 days) for both groups. The mortality rate was 0% for the first group and 3% (1 patient died of myocardial infarction and one of postoperative hemorrhage) for the anastomotic one. The morbidity rate was 24% in the occlusion group whereas 32% in the latter. Pancreatic fistula was observed in 13 patients (23.2%) in the anastomotic group and in 2 patients (5.4%) in the occlusion group, and they were all managed conservatively. Pancreatic fistula was defined according to the International Study Group of Pancreatic Fistula guidelines [5], as amylase rich fluid (with an amylase concentration in the drainage fluid more than 3 times the serum concentration). The fluid was collected by needle aspiration from an intra-abdominal collection or from the intraoperatively placed drain on the third postoperative day. Other complications included 2 wound infections in group one (5.4%) and 3 (5.3%) in group two, 1 bile leak (2.7%) in group one and 2 (3.5%) in group two, 3 cases of delayed gastric emptying (8.1%) in group one and 2 (3.6%) in group two, and 3 urinary tract infections in group one (8.1%) and 4 (7.1%) in group two (Table 2).

Finally, no difference regarding one year survival rates was recorded.

According to the literature, the function of the Langerhans' islets is not affected by pancreatic duct occlusion. In our series, there was no difference between the two groups regarding patient's needs for pancreatic enzymes replacement or the development of postoperative diabetes. In general no chronic abdominal pain and chronic pancreatitis differences were detected between the two groups (Table 3).

## 4. Discussion

During the last two decades PD is used with increasing frequency for the management of malignant, and also for benign periampullary lesions. Postoperative mortality rate has decreased significantly in comparison to the previous years and is reported below 5% in high volume centers [6], but morbidity is still considered high. Anastomotic leakage at the pancreaticojejunostomy is one of the most severe complications of PD with 5–25% rate among series according to different definitions [7, 8], leading to prolonged hospital stay, interventional radiology procedures, relaparotomy, and high mortality rates. Many anastomotic techniques have been described in order to prevent leakage from the pancreatic anastomosis, but unfortunately with no uniform results [9]. 

An interesting approach to this problem is the occlusion of the pancreatic remnant after PD. The absence of the pancreaticojejunal anastomosis could prevent a large portion of the postoperative complications of pancreatic surgery. Occlusion can be performed either by suturing the pancreatic duct with nonabsorbable suture material or by using synthetic materials, like fibrin glue solution, Tissucol, and Neoprene. Moreover, it has been reported that a pancreatic fistula resulting from an oversewn pancreatic remnant is less dangerous than one from a pancreaticojejunal anastomosis because there is no defect of the small bowel, which could lead to activation of leaking pancreatic enzymes [10]. 

In our series, pancreatic fistula rate was significantly lower in the pancreatic occlusion group, and the ones which occurred were managed with prolonged pancreatic drainage, indicating a low grade fistula. The most significant anatomic features influencing the development of this complication are the consistency of the pancreatic parenchyma and the size of the pancreatic duct [11]. These factors appeared to be similar in both of our groups. Furthermore, if both of the preoperative imaging studies (ERCP and MRCP) and the intraoperative findings indicate a dilated duct, then the pancreatic remnant occlusion technique seems to be a rather interesting alternative option. Despite the classic knowledge that dilated ducts indicate easier and more durable duct-to-mucosa pancreaticojejunal anastomosis, in our series dilated ducts in fibrotic pancreas with spurt of the pancreatic fluid at the time of opening of the pancreatic duct mean an already occluded duct, which we occlude once again after pancreatectomy by stapling and oversewing. 

Pancreatic remnant occlusion is a safe, less complicated alternative management of the pancreatic stump during Whipple's procedure. Additionally, it neither affects the oncologic principles of the surgical procedure nor the long-term survival of patients treated for cancer of the head of the pancreas. 

## 5. Conclusions

Pancreatic remnant occlusion is a safe, time consuming, and less complicated alternative management of the pancreatic stump during Whipple's procedure. Additionally, it does not affect the oncologic principles and long-term survival of patients treated for cancer of the head of the pancreas. 

## Figures and Tables

**Table 1:** Indications for surgery (common bile duct (CBD))*.

	Group 1 [*n* (%)]	Group 2 [*n* (%)]
Pancreatic cancer	26 (70.27)	34 (60.7)
Ampulla of Vater carcinoma	8 (21.62)	12 (21.4)
CBD carcinoma*	1 (2.7)	6 (10.7)
Chronic pancreatitis	2 (5.4)	4 (7.1)

**Table 2:** Postoperative complications.

	Group 1 [*n* (%)]	Group 2 [*n* (%)]
Pancreatic fistula	2 (5.4)	13 (23.2)
Wound infection	2 (5.4)	3 (5.3)
Bile leak	1 (2.7)	2 (3.5)
Delayed gastric emptying	3 (8.1)	2 (3.5)
Urinary tract infections	3 (8.1)	4 (7.1)

**Table 3:** Long-term outcome.

	Group 1 [*n* (%)]	Group 2 [*n* (%)]
Diabetes mellitus	10 (27)	14 (25)
Exocrine insufficiency	12 (32.4)	12 (21.4)
Weight loss	8 (21.6)	13 (23.2)
Chronic abdominal pain	1 (0.2)	2 (0.3%)
Development of chronic pancreatitis	1 (0.2)	1 (0.1)
